# On the Reflex Function of the Medulla Oblongata and Medulla Spinalis

**Published:** 1834-04-01

**Authors:** 


*On the Reflex Function of the Medulla Oblongata and Medulla Spinalis.* By MARSHALL HALL, M.D., F.R.S. L. & E., &c. [From the *Philosophical Transactions.*]—London, 1833. 4to. pp. 31.

IN this paper Dr. Hall announces a discovery, which he thinks he has made, of a power resident in the medulla oblongata and spinal cord, not hitherto recognised by physiologists, and to whose action he applies the name of the *reflex function.*

As we shall have occasion to show, in the sequel of this article, that Dr. Hall has greatly misconstrued the opinions of other physiologists, we must be careful not to fall into the same sin ourselves; we shall therefore, at the risk of being thought somewhat prolix in our extracts, introduce our author's general exposition of his subject in his own words.

After alluding to the experiments of some modern physiologists on the medulla oblongata and spinal cord, Dr. Hall gives the following account of the property to which he wishes to direct attention.

"This property is characterized by being excited in its action, and *reflex* in its course; in every instance in which it is exerted, an impression made upon the extremities of certain nerves is conveyed to the medulla oblongata or the medulla spinalis, and is reflected along other nerves to parts adjacent to, or remote from, that which has received the impression. It is by this reflex character that the function to which I have alluded is to be distinguished from every other. There are, in the animal economy, four modes of muscular action, of muscular contraction. The *first* is that designated *voluntary;* volition, originating in the cerebrum, and spontaneous in its acts, extends its influence along the spinal marrow and the motor nerves, in a *direct line*, to the voluntary muscles. The *second* is that of *respiration:* like volition, the motive influence in respiration passes in a *direct line* from one point of the nervous system to certain muscles; but as voluntary motion seems to originate in the cerebrum, so the respiratory motions originate in the medulla oblongata. Like the voluntary motions, the motions of respiration are spontaneous; they continue at least, after the eighth pair of nerves has been divided. The *third* kind of muscular action in the animal economy is that termed *involuntary:* it depends upon the principle of irritability, and requires the *immediate* application of a stimulus to the nervo-muscular fibre itself. These three kinds of muscular motion are well known to physiologists; and I believe they are all which have been hitherto pointed out. There is, however, a *fourth*, which subsists, in part, after the voluntary and respiratory motions have ceased, by the removal of the cerebrum and medulla oblongata, and which is attached to the medulla spinalis, ceasing itself when this is removed, and leaving the irritability undiminished. In this kind of muscular motion, the motive influence does not originate in any central part of the nervous system, but at a distance from that centre; it is neither spontaneous in its action, nor direct in its course; it is, on the contrary, *excited* by the application of appropriate stimuli, which are not, however, applied immediately to the muscular or nervo-muscular fibre, but to certain membranous parts, whence the impression is carried to the medulla, *reflected*, and reconducted to the part impressed, or conducted to a part remote from it, in which muscular contraction is effected. The first three modes of muscular action are known only by actual movements or muscular contractions. But the reflex function exists as a continuous muscular action, as a power presiding over organs not actually in a state of motion, preserving in some, as the glottis, an open, in others, as the sphincters, a closed form, and in the limbs, a due degree of equilibrium, or balanced muscular action,—a function, not, I think, hitherto recognised by physiologists. The three kinds of muscular motion hitherto known may be distinguished in another way. The muscles of voluntary motion and of respiration may be excited by stimulating the nerves which supply them, in any part of their course, whether at their source, as a part of the medulla oblongata or medulla spinalis, or exterior to the spinal canal: the muscles of involuntary motion are chiefly excited by the actual contact of stimuli. In the case of the reflex function alone, the muscles are excited by a stimulus acting mediately and indirectly in a curved and reflex course, along superficial subcutaneous or submucous nerves proceeding to the medulla, and muscular nerves proceeding from the medulla. The first three of these causes of muscular motion may act on detached limbs or muscles. The last requires the connexion with the medulla to be preserved entire.

"All the kinds of muscular motion may be unduly excited. But the reflex function is peculiar in being excitable into modes of action not previously subsisting in the animal economy, as in the cases of sneezing, coughing, vomiting, &c. The reflex function also admits of being permanently diminished or augmented, and of taking on some other morbid forms, of which I shall treat hereafter. I shall thus have occasion to speak of the reflex function as the source of equilibrium in the musculur system; as excitable into various actions, which, however familiar, are not constant; and as assuming morbid forms. Before I proceed to the detail of the experiments upon which this disquisition rests, it may be well to point out several instances in illustration of the various sources and modes of muscular action which have been enumerated. None can be more familiar than the act of swallowing, yet how complicated is this act! The apprehension of the food by the teeth, the tongue, &c., is voluntary, and cannot therefore take place in an animal from which the cerebrum is removed. The transition of the food over the glottis and along the middle and lower parts of the pharynx depends upon the reflex function; it can take place in animals from which the cerebrum has been removed, or the ninth pair of nerves divided; but it requires the connexion with the medulla oblongata to be preserved entire; and the actual contact of some substance which may act as a stimulus: it is attended by the accurate closure of the glottis, and by the contraction of the pharynx. The completion of the act of deglutition is dependent upon the stimulus immediately impressed upon the muscular fibres of the œsophagus, and is the result of excited irritability.

"The example which I have given is one of excited reflex function. The condition of the glottis during respiration, and that of the pharynx and of the sphincters, at all times, except during the acts of deglutition or of excretion, afford equally interesting and familiar examples of the permanent influence of that function. Whilst the nervous connexion between the larynx and the medulla oblongata is preserved entire,—in the rabbit (*Lepus cuniculus*), for example,—the glottis is preserved open, being slightly dilated during each act of inspiration; but, if the superior laryngeal nerves be divided, the aperture immediately becomes so much diminished, that a state of excessive dyspnœa is induced. The sphincter ani, on the other hand, remains closed in the decapitated turtle (*Chelonia mydas*), if the lower part of the medulla spinalis be left in its canal; but it becomes immediately relaxed and open, if this part of the nervous system be withdrawn. The action of this muscle depends upon the medulla spinalis, and not upon the brain only.

"However plain these observations may have made the fact, that there is a function of the nervous and muscular system distinct from sensation, from the voluntary and respiratory motions, and from irritability, it is right, in every such inquiry as the present, that the statements and reasonings should be made with the experiment, as it were, actually before us. It has already been remarked, that the voluntary and respiratory motions are spontaneous acts, not necessarily requiring the agency of a stimulus. If, then, an animal can be placed in such circumstances that such motions will certainly not take place, the power of moving remaining, it may be concluded that volition, and the motive influence of respiration, are annihilated. Now, this is effected by removing the cerebrum and the medulla oblongata. These facts are fully proved by the experiments of Le Gallois and M. Florens, and by several which I proceed to detail, for the sake of the opportunity afforded by doing so of stating the argument most clearly. I divided the spinal marrow of a very lively snake (*Coluber natrix*,) between the second and third vertebræ. The movements of the animal were, immediately before, extremely vigorous and unintermitted. From the moment of the division of the spinal marrow, it lay perfectly tranquil and motionless, with the exception of occasional gaspings and slight movements of the head. It became quite obvious that this state of quiescence would continue indefinitely, were the animal secured from all external impressions. Being now stimulated, the body began to move with great activity, and continued to do so for a considerable time, each change of position or situation bringing some fresh part of the surface of the animal into contact with the table or other objects, and renewing the application of stimulus. At length the animal became again quiescent; and, being carefully protected from all external impressions, it moved no more, but died in the precise position and form which it had last assumed. It requires a little manœuvre to perform this experiment successfully: the motions of the animal must be watched, and slowly and cautiously arrested by opposing some soft substance, as a glove or cotton-wool; they are by this means gradually lulled into quiescence. If at this moment the figure last assumed be sketched upon paper, and the animal be left, protected from external impressions, it will be found to retain the same identical form when all vitality has ceased. The slightest touch with a hard substance, the slightest stimulus, will, on the other hand, renew the movements of the animal in an active form. But that this phenomenon does not depend upon sensation is further fully proved by the facts, that the position last assumed, and the stimuli applied, may be such as would be attended by extreme or continued pain, if the sensibility were undestroyed: in one case, the animal remained partially suspended over the acute edge of the table; in others, the infliction of punctures, and the application of a lighted taper, did not prevent the animal, still possessed of active powers of motion, from passing into a state of complete and permanent quiescence. The same observations were made upon various other animals,—the turtle, the viper (*Vipera Berus*), the toad (*Bufo vulgaris*), the frog (*Rana temporaria*), the eft (*Triton cristatus*), &c. It may therefore be stated as a general fact, that if an animal be deprived of the cerebrum and medulla oblongata, and placed under an inverted bell-glass, or otherwise protected from external stimuli, it will not move, however easily it may be excited to motion by external impressions. I must now solicit the attention of the Society to three important points: It is obvious, 1st, that sensation can act, in inducing muscular motion, only through the medium of volition; 2dly, that, in the experiments which have been described, volition, the *will*, and not the *power*, to move, was annihilated; 3dly, that in such cases (volition being destroyed, and the agency of sensation excluded,) the influence of external impressions, which might be supposed to induce pain, must have been exerted upon some property of the nervous system different from sensibility."

Dr. Hall admits that "many of the facts which depend upon the reflex function have long been known to physiologists," but he maintains that "these facts only extend to the excited action of the reflex function seen in the limbs; and even they have been erroneously ascribed to sensation and volition, or instinct."

Now, Dr. Hall has fallen into a very unaccountable error, in supposing that the three first kinds of muscular action which he enumerates, the *voluntary*, the *respiratory*, and the *involuntary from excited irritability*, are the only ones hitherto recognised by physiologists; for writers on this science have long been accustomed to refer a variety of phenomena, and among others muscular contraction, to what they have termed *nervous sympathy*, constituting a fourth source, different from any of the former; and which, as viewed by some authors whom we shall presently quote, appears to be, in *principle*, altogether similar to the *reflex function*.

We may cite the following passage from Haller, to prove that a cause of muscular action, not referable to the classes called voluntary, involuntary, or respiratory, has been already very distinctly recognised.

"Motus sympathici alii ex presente ad organum stimulo oriuntur; alii *a stimuli in cerebro vestigio*. Illius quidem generis exemplum est in colica nephritica, quando vomitus cietur occasione stimuli ad ureterem fixi. Eadem hic fiunt, quæ alibi in majoribus nervorum solicitationibus, nempe convulsio a proprio nervo ad vicinos, et connexos nervos, et demum ad omnes, spargitur quando fortissima est. *Nullum hic voluntatis vestigium*, sed necessarius nervi a nervo irritati consensus." (*Element. Physiolog.*, lib. xi. sect. iii. § ix.)

We would ask whether the second set of muscular actions here alluded to by Haller are referred by him, or *could* be referred by any body, to the voluntary class, to the respiratory, or to the involuntary from excited irritability?

We know not, in fact, a single physiologist, from Willis, who first pointed out the nerves as the cause of sympathetic motion, to Alison, the ingenious expositor of Whytt's doctrine, who has failed to recognise *nervous sympathy* as a source of muscular action not dependent on volition, excited irritability, or the respiratory function. We maintain further, that the doctrine of nervous sympathy has, in the hands of certain physiologists, assumed a form altogether similar to the *reflex function* of Dr. Marshall Hall. We beg the attention of the reader to the following extract from Whytt's treatise on the Sympathy of the Nerves. This author, after enumerating a great variety of sympathetic sensations and motions, and demonstrating very satisfactorily that they cannot depend on the anastomosis of nerves, proceeds as follows:

"If, therefore, the various instances of sympathy cannot be accounted for from any union or anastomosis of the nerves in their way from the brain to the several organs, and if there are many remarkable instances of consent between parts whose nerves have no connexion at all, it follows that all sympathy must be referred to *the brain itself and spinal marrow*, the source of all the nerves. But, for a more direct proof of this, we may observe, that the consent of the several parts instantly ceases when their communication with the origin of the nerves is interrupted. Thus, though the muscular coat of the stomach, in an animal newly dead, is excited into contraction by irritation, yet the diaphragm is no way affected by this stimulus. In like manner, when any of the muscles of the leg of a frog are pricked, most of the muscles of the legs and thighs contract, *even after cutting off its head, if the spinal marrow be left entire;* but, when that is destroyed, although the fibres of the stimulated muscle are affected with a weak tremulous motion, yet the neighbouring muscles remain wholly at rest. Farther, the effects of pain, and of fear and other passions, in preventing several sympathetic motions, seem to shew that the cause of that consent which obtains between the parts of animals is to be referred to the *origin of the nerves;* and, since certain affections of the mind, excited by the action of external objects on the organs of sense, produce extraordinary motions and other effects on the body, merely by affecting the brain, why may not impressions made on the nerves in other parts produce likewise, through the intervention of the brain, various motions and other effects in distant parts of the body? The analogy is obvious. Lastly, notwithstanding the many sympathetic motions which are daily observed by physicians to arise from an irritation of the nerves in different parts of the body, yet, when the nerve going to any muscle is irritated, there is no motion excited in any part, except in the muscle to which it is distributed. Does it not hence appear highly probable, that the various sympathetic motions of animals produced by irritation, whether in a sound or morbid state, are owing not to any union or connexion of their nerves, but to particular sensations excited in certain organs, and thence communicated to the brain or spinal marrow?" (*Works,* p. 510, Edin. 1768.)

Whytt maintained that, in all these instances of sympathetic motion, a certain sensation is excited, in consequence of which a motory impulse is communicated to the muscle; and there is no doubt that, in the greater part, there is a very manifest sensation, whether this be regarded as the cause of the motion or not: but it is very evident that Whytt did not consider the process as necessarily connected with sensation, and consequent volition, in the ordinary acceptation of these terms; because he distinctly states, that in many cases both the sensation and the exertion of the mind in producing the sympathetic motion are *unattended with consciousness;* and the whole of the eleventh section of his treatise on the Vital and Involuntary Motions is occupied in shewing how an action which appears to be, and is involuntary, may nevertheless be the result of an obscure and transitory impression on the mind. In this opinion Whytt has been recently supported by Professor Alison, (*Ed. Medico-Chirurg. Transact.*, vol. ii.) who, grounding his analogy on the indisputable fact, that by far the greater number of such sympathetic motions are actually accompanied with sensation, argues that in all cases a sensation is excited in the mind, although it be in many too transient to awaken the consciousness of the individual: he adds, however, that we have no means of judging "whether it is, strictly speaking, from mental sensations that the different sympathetic phenomena proceed, or whether they are more properly the result of those physical changes in the nervous matter which immediately precede and cause the sensations, and which are known to us only through them."

This question of sensations mooted by Whytt evidently appertains rather to metaphysics than to physiology, and hinges, in a great measure, on the precise meaning attached to the word *sensation*. It is moreover a question which, being alike removed from the sphere of consciousness and of experiment, is not very likely ever to be settled, and is hence of little importance to the practical physiologist.

Let us see wherein Whytt and Dr. Hall agree, and wherein they differ. They agree that an impression made on the extremity of one nerve is propagated to the origin of that nerve, and thence reflected in the course of another nerve to a muscle, where it occasions contraction; which contraction the individual can neither excite nor prevent by an effort of his will. They differ in this: Whytt thinks that there is, in the process alluded to, an intervention of the mind, which intervention, since it is unattended with consciousness, he cannot prove. Dr. Hall denies such intervention, but nevertheless cannot demonstrate that it does not take place. Dr. Hall, then, is surely not justified in imputing ignorance of the *reflex function* to Whytt, who was as well acquainted with the fact of its existence as himself, merely because that author attempted the solution of the phenomena by a metaphysical hypothesis, which no man living can either prove or disprove. On the same principle, Whytt might be made out ignorant of the action of the heart, since he believed that this also took place by the intervention of the mind: he might be made out ignorant of every vital phenomenon dependent on nervous influence, for he ascribed them all to these *mental sensations*,—a doctrine which, we confess, is to us utterly unintelligible, but which has decidedly no relation to any one practical point in physiology.

With regard to the assertion that the phenomena of the reflex function have only been observed in the limbs, the reader has but to refer to the works of Whytt, and the paper of Dr. Alison already quoted, to be convinced of its inaccuracy.

We believe, then, we are justified in the conclusion that the reflex function of Dr. Hall differs in no essential point from the nervous sympathy of Whytt and Alison, except in its restriction to the medulla oblongata and spinal cord, to the exclusion of the brain; which exclusion, however, as we shall presently show, is not warranted by facts.

Dr. Hall imputes, with infinitely greater justice, the same error to Legallois as to Whytt,—that of ascribing the motions resulting from the reflex function to volition; and there is no doubt that M. Legallois has, in one instance, actually fallen into this mistake. There is, in the works of this physiologist, so inexplicable a confusion in all that relates to voluntary motion, that one is almost inclined to suppose that he does not know the meaning of the word *voluntary*, since he tells us very distinctly in one place that the brain is the sole organ of the *will*,* and as distinctly in another place that *voluntary* motion may occur in a decapitated animal.† Verily the wisdom of this world is foolishness; for we have here a distinguished physiologist, after much laborious research, and flat self-contradiction, arriving at the conclusion that an animal can will after its head is cut off!—a conclusion which a plain man, who knew nothing about physiology, would at once pronounce to be ridiculous, and with much reason. We wish physiologists would take common sense along with them!

To return from this digression. Having shewn that there is nothing new or original in the *principle* of the reflex function, it is time to lay before the reader the experiments from which Dr. Hall deduces his various conclusions respecting it.

"The first experiment which I made was upon the turtle. This animal was decapitated in the manner usual with cooks, by means of a knife, which divided the second or third vertebra. The head being placed upon the table for observation, it was first remarked that the mouth opened and shut, and that the submaxillary integuments descended and ascended alternately from time to time, replacing the acts of respiration. I now touched the eye or eyelid with a probe. It was immediately closed: the other eye closed simultaneously. I then touched the nostril with the probe. The mouth was immediately opened widely, and the submaxillary membranes descended. This effect was especially induced on touching the nasal fringes situated just within the anterior part of the maxilla. I passed the probe up the trachea, and touched the larynx. This was immediately followed by a forcible convulsive contraction of the muscles annexed to it. Having made and repeated these observations, I gently withdrew the medulla and brain. All the phenomena ceased from that moment. The eye, the nostril, the larynx, were stimulated, but no movement followed.

* Expériences sur le Principe de la Vie. Avant propos, p. 3. † Idem, p. 11.

"The next observations were made upon the other parts of the animal. The limbs, the tail, were stimulated by a pointed instrument, or a lighted taper. They were immediately moved with rapidity. The sphincter was perfectly circular and closed; it was contracted still more forcibly on the application of a stimulus. The limbs and the tail possessed a certain degree of firmness, or tone, recoiled on being drawn from their position, and moved with energy on the application of the stimulus. On withdrawing the spinal marrow gently out of its canal, all these phenomena ceased. The limbs were no longer obedient to stimuli, and became perfectly flaccid, having lost all their resilience. The sphincter lost its circular form and its contracted state, becoming lax, flaccid, and shapeless. The tail was flaccid, and unmoved on the application of stimuli.

"This experiment affords evidence of many important facts in physiology. It proves that the presence of the medulla oblongata and spinalis is necessary to the contractile function of the eyelids, the sub-maxillary texture, the larynx, the sphincters, the limbs, the tail, on the application of stimuli to the cutaneous surfaces or mucous membranes. It proves the reflex character of this property of the medulla oblongata and spinalis, and the dependence of these motions upon the reflex function. It proves that the tone of the limbs, and the contractile property of the sphincter, depend upon the same reflex function of the medulla spinalis, effects not hitherto suspected by physiologists. I must now state that the phenomena which have been detailed subsist in distinct portions of the divided nervous system. If, after severing the head of the turtle, the lower extremities and the tail be separated together, in the manner usual with cooks, the phenomena which I have described are still observed in the distinct and separate portions of the animal. The head, the anterior extremities, and the tail, present the movements which have been described, when severally stimulated. The posterior extremities alone were observed to be flaccid and unimpressible by stimuli; and these were found, on examination, to have been separated from their connexion with the spinal marrow. An interesting experiment demonstrates the powerful influence of the reflex function over the sphincter ani in the turtle. If, after the removal of the tail and the posterior extremities, with the rectum, and of course with a portion of the spinal marrow, water be forced into the intestine, by means of Read's syringe, both the cloaca and the bladder are fully distended before any part of the fluid escapes through the sphincter, which it then does on the use of much force only, and by jerks. The event is very different on withdrawing the spinal marrow: the sphincter being now relaxed, the water flows through it at once in an easy continuous stream, with the application of little force, and without inducing any distention, even of the cloaca. I was first struck with the phenomena of the reflex function of the spinal marrow in the separated tail of an eft. On being excited by the point of a needle passed lightly over its surface, it contracted and moved as if it still formed a part of an entire animal. On another occasion, having removed the head of a frog, I divided the spine between the third and fourth vertebræ, and separated the upper portion of the animal from the lower. There were then the head, the anterior extremities, and the posterior extremities, with their corresponding portions of medulla, as three distinct parts of an animal. Each preserved the reflex function. On touching an eye, it was retracted, and the eyelids closed, whilst similar phenomena were observed simultaneously in the other eye. On removing the medulla, these phenomena ceased. On pinching the toe of one of the anterior extremities, the limb and the opposite limb equally moved. On removing the spinal marrow, these phenomena also ceased. Precisely similar effects were observed in regard to the posterior extremities. Similar phenomena are also observed in the snake. If the head be removed, and a pointed instrument, or a lighted taper, be brought into contact with any part of the surface, it is instantly moved. The motion consists in a flexion of the entire part, and in a concentric movement of the integuments towards the point irritated; so that the muscles situated along the spine, and certain muscles analogous to the panniculus carnosus, are excited to contraction. The extremity of the tail is most impressible. The function which presides over these movements subsisted in every part of the animal separated from the rest, but instantly ceased on removing the spinal marrow. On touching a point immediately within the teeth of the upper jaw, the larynx was suddenly drawn downwards and closed. These movements could also be excited by touching the nostrils. They ceased on removing the medulla oblongata. Similar phenomena are seen also in the very young of the mammalia. A rabbit, one day old, was immediately deprived of all voluntary or respiratory motion, with the exception of gaspings, by dividing the spinal marrow near the occiput. Yet the head and the limbs moved, on stimulating the ears or the feet. These movements ceased in a quarter of an hour, but were renewed by an artificial respiration. The phenomena were precisely similar after decapitation, hæmorrhage being prevented and artificial respiration maintained. All ceased on removing the medulla oblongata and spinalis. One of the most remarkable of the phenomena attached to the reflex function in animals, is that presented by those muscles of the hedgehog (Erinaceus Europæus) by means of which that animal assumes, in certain circumstances, the form and firmness of a ball. The reflex function seems especially to connect the roots of the spines with the muscles. If the animal be examined under the influence of hybernation, the reflex function continues for some hours after the brain is removed; the panniculus carnosus, the limbs, the tail, the larynx, the sphincter ani, remain excitable, and retain a degree of tone. These phenomena cease on removing the medulla spinalis. The phenomena of the reflex function seen in the panniculus carnosus, and in other muscles of the hedgehog, are also particularly displayed in the very young animal, in which the peculiar movements of this creature are excitable for a considerable time after decapitation, or the division of the spinal marrow, and long after the cessation of the voluntary and respiratory motions, when it is in a languid and dying state. In the case of the decapitated young hedgehog, after all gasping had ceased, motions of the larynx are still excited on irritating the nostrils, or on irritating the medulla itself; just as the peculiar motions of the trunk are excited on irritating the limbs, tail, or spines, or the spinal marrow."

Dr. Hall corroborates these experiments by a reference to some cases of anencephalous children, which afford the same data for reasoning, with the great advantage of the absence of all mutilation; a circumstance which throws infinite uncertainty over most experiments of this kind on living animals. We hold one anencephalous child to be worth at least a thousand decapitated frogs or dogs. The cases instanced by Dr. Hall have already attracted much and deserved attention from physiologists; we need not therefore detail them here.

Now, on reviewing all these experiments, the first question that suggests itself is, on which of them does Dr. Hall ground his restriction of the reflex function to the medulla oblongata and spinal marrow? The experiments unquestionably shew that the said function *does* exist in these parts: but where is the proof that it *does not* exist in the brain?

The only experiments which can be conceived to bear in any degree upon the question are those made on the eyes of the turtle and the frog. Whether it be from these that our author draws his conclusions or not, we cannot tell; but we presume so, because none of the others could even be supposed to have any relation to the matter. We must then remark of the first of these two experiments, that, as the brain and medulla oblongata were both removed at the same time, it cannot prove anything with reference to the brain exclusively; to say nothing of the fact, that the anatomy of the nerves distributed to the appendages of the eye in this class of animals is not sufficiently known to enable us to say with precision, from what source they are derived; and it is evident that the only nerves through which the reflex power of the brain can be brought to the test are those which are known to be *cerebral* nerves.* The latter objection applies

* Thus, if the muscles which close the eyelids be supplied, as the *orbicularis* in man, by the *portio dura*, this is a very improper nerve through which to illustrate any function of the brain; since, though it be nominally a cerebral nerve, there is every reason to believe that it is intimately connected, both in its origin and function, with the respiratory nerves; and its function will therefore be necessarily destroyed by the removal of the medulla oblongata.

with equal force to the experiment on the head of the frog; the nerves which supply the eyelids, and the mechanism by which the apparent retraction of the eye is effected, having not hitherto, as far as we know, attracted the particular attention of anatomists. It is to be observed also, that, although the effect of withdrawing the medulla was obvious, the effect of withdrawing the brain, and leaving the medulla *in situ*, was not tried. If it were only on this ground, therefore, the experiment is inconclusive. But the truth is, that, from the close connexion of the medulla oblongata with the base of the brain, it is nearly impossible to draw any conclusion with regard to the respective functions of these parts from experiments in which one of them is withdrawn. A function which is in reality exercised by the brain may, nevertheless, be annihilated by the removal of the medulla, and *vice versâ*. It is, however, unnecessary to have recourse to experiments of a kind which at best are liable to numerous fallacies, for the solution of a question which daily observation sufficiently elucidates. It is very plain that the reflex function *does* exist in the brain. When, for example, the pupil contracts in consequence of the impression of light on the retina, we have every condition of the reflex function. An impression made on the optic nerve is reflected along the nerves of the iris to the latter organ, where it occasions a contraction which is independent of the will; and the brain must here be the reflecting centre, because all the nerves concerned in the process are cerebral nerves. The impression of light, also, on the retina of one eye, affects sympathetically the iris of the other, as we often observe in those cases where only one eye is affected with amaurosis. It will not, we presume, be objected to this example, that it is doubtful whether the iris be *muscular* or not, because this question may now be considered as nearly settled in the affirmative;* and whether it be so or not, does not in reality affect the question of the reflex power exercised by the brain.

If, however, this objection should be made, we will adduce another example, which is free from it, and will answer our purpose as well. It is a familiar fact, that when the supraorbitary nerve is irritated, as by neuralgia, or any other cause, the adjacent muscles are affected with spasms, which spasms cease on the division of the nerve. Now, if this be not an instance of reflex action in the brain, what is it?

Other examples might be adduced, but, as these are less unequivocal, we pass them over. If it be not easy to multi-

* Vide Bostock's Physiology, vol. i. p. 182.

ply instances of the reflex power of the brain, it is because comparatively few muscles are supplied by cerebral nerves.

Our author's conclusions with respect to the glottis and the sphincter muscle are evidently premature: analogy may render it not improbable that the action of these parts is influenced by the reflex function of those portions of the nervous mass from which they respectively derive their nerves; but the logical deduction from the experiments is merely that the action of the glottis is dependent on some function of the medulla oblongata, and that of the sphincter on some function of the spinal marrow.

The same remark applies to the conclusion with respect to the panniculus carnosus.

The facts relating to the integrity of the reflex function in the several insulated portions of the nervous mass which fill the spinal canal are new and interesting, and they appear to us to constitute the only *discovery* contained in this paper. It is to be observed, however, that the inference is hitherto valid only with respect to cold-blooded animals.

The view taken by Dr. Hall of the reflex function as the cause of the tone and equilibrium of the muscles, is ingenious and worthy of attention: still, however, it is only an hypothesis; and we think that the *equilibrium* of the muscles may be much better explained on the doctrine of the *muscular sense.* With reference to their *tone,* one explanation is nearly as good as another, because the very existence of this property is hypothetical.

We have not space to follow our author in the various applications which he makes of the reflex function to the illustration of several morbid phenomena; but, if the reader will refer to them, we think he will consider us justified in affirming, that some of them are hypothetical, and that others have been repeatedly made by former writers, and are indeed generally familiar to pathologists.

In considering the reflex function, especially with reference to disease, it would be interesting to inquire how far it may be exercised by the ganglia of the great sympathetic nerve; but we have not at present time for the investigation, which would also be rendered difficult by our imperfect knowledge of the functions and real connexions of this remarkable nerve. Dr. Copland, in the learned and excellent notes appended to his translation of Richerand's Physiology, divides nervous sympathies into "the *reflex*, and the *direct:* the former arising through the instrumentality of the sensorium, the latter taking place independently of it, through the means of the ganglial nerves, and chiefly of those which are distributed to the blood-vessels, and which form communicating chords between the viscera." (P. 546.) It is possible, however, as hinted above, that one of the uses of the ganglia may be to act as reflecting centres, by which stimuli applied to the extremity of one nerve are propagated to those of others; in which case, the term *reflex* may be applicable also to the sympathetic actions of the ganglionic system.

In concluding our review of Dr. Hall's paper, we feel ourselves bound to state, that, although it contains some ingenious reasoning, and is well worthy of perusal, [we never read anything of our author's that was not,] its claims to originality appear to us to be exceedingly small.

